# Recurring pain in right iliac fossa in children: report of two cases associated with food allergies

**DOI:** 10.1186/1939-4551-8-S1-A278

**Published:** 2015-04-08

**Authors:** Isaac Azevedo Tenorio, Selma Sabra, Aderbal Sabra

**Affiliations:** 1 Unigranrio, Brazil

## Objective

To present two cases of food allergy with unusual presentation and discuss the diagnostic approach in children

## Cases

*Case # 1:* Girl, 11, afflicts right lower quadrant pain for 3 months. Background

Prematurity, atopy (dermatitis, rhinitis, colic). Sister and atopic mother. Physical examination: exquisite tenderness in the right iliac fossa, bilateral Cord colic pain. Dry skin. Laboratori data:all hematology is normal. Examination of normal urine, serial parasitological (-). EDN (eosinophil derived neurotoxin) fecal> 3210 ng / ml (VN <360NG / ml). Colonoscopy:ILNH, ileal lymphoid nodular hyperplasia. Food allergy Score: 9

*Case No. 2:* Boy, 9 years. Pain in right lower quadrant , abdominal pain for three months located in the right iliac fossa, a predominantly nocturnal and radiating to right thigh. A child psychiatrist prescribes antidepressants. Personal Background

Mixed Breastfeeding from birth. Atopy: asthma, atopicdermatitis, infantcolic. Family history: Mother allergic to foods, presents father rhinitis. Physical examination: bilateral colic Rope. Tenderness in the right iliac fossa. Lab: Immunoglobulin E 160.5 IU / mL (normal <90) Colonoscopy lymphoid hyperplasia in distal ileum. Score of foodallergy: 11.

Both cases improve with hypoallergenic diet.

## Conclusion

The presence of lymphoid nodular hyperplasia and family and / or personal history of atopy severe gastrointestinal symptoms may be due to food allergy. The elimination diet is diagnostic and therapeutic turn.

## Consent

Written informed consent was obtained from the patients for publication of this abstract and any accompanying images. A copy of the written consent is available for review by the Editor of this journal.

